# SARS-CoV-2 Antibodies in Hemodialysis Patients Six Months after Infection Compared to Healthcare Workers

**DOI:** 10.1155/2021/4747221

**Published:** 2021-12-01

**Authors:** Henri Boulanger, Salima Ahriz Saksi, Jedjiga Achiche, Florence Batusanski, Nicolas Stawiarski, Ali Diddaoui, Luc Fromentin, Mokhtar Chawki

**Affiliations:** ^1^Department of Nephrology and Dialysis, ELSAN, Clinique de l'Estrée, 35 Rue d'Amiens, 93240 Stains, France; ^2^Medical Analysis Laboratory, Biogroup, 40 Rue du Bois Moussais, 93240 Stains, France; ^3^Medical Analysis Laboratory, Clinique Claude Bernard, 9 Avenue Louis Armand, 95120 Ermont, France; ^4^Department of Nephrology and Dialysis, Clinique Claude Bernard, 9 Avenue Louis Armand, 95120 Ermont, France

## Abstract

**Background:**

The humoral response to SARS-CoV-2 infection in hemodialysis patients needs to be clarified.

**Methods:**

In this retrospective study performed in two dialysis facilities, we measured the circulating levels of SARS-CoV-2 antibodies in patients who were on maintenance hemodialysis during the first wave of the epidemic in March and April 2020 and were still alive 6 months later. We also investigated associations between the patients diagnosed as infected during the first wave and several clinical, biological, and radiological parameters of COVID-19. Finally, we compared these circulating levels of SARS-CoV-2 antibodies with those of a control group of healthcare workers infected during the same period.

**Results:**

Of the 299 hemodialysis patients who recovered from the first wave of the epidemic 6 months before, 59 had a positive SARS-CoV-2 antibody whereas only 45 patients were diagnosed as infected during the first wave of the epidemic. All infected hemodialysis patients developed circulating antibodies. Using a clustering method, a significant correlation was identified between the cluster with the lowest circulating levels of SARS-CoV-2 antibodies and the severity of COVID-19 based on several parameters including CRP, BNP, lymphocyte count, neutrophil-lymphocyte ratio, and oxygen requirements, as well as pulmonary involvement on chest scan. Moreover, the circulating levels of the SARS-CoV-2 antibodies in surviving hemodialysis patients (*n* = 59) were similar to those of the control group (*n* = 17).

**Conclusion:**

The main finding of this study is that all of the surviving hemodialysis patients who were diagnosed with SARS-CoV-2 infection from March to April 2020 developed a persistent humoral response with significant circulating levels of SARS-CoV-2 antibodies, 6 months later. Another important finding is that surviving hemodialysis patients who had more severe disease had lower circulating levels of SARS-CoV-2 antibodies. Finally, circulating levels of SARS-CoV-2 antibodies were similar in surviving hemodialysis patients and healthcare workers without kidney disease.

## 1. Introduction

The development of sustained antibodies in response to Severe Acute Respiratory Syndrome Coronavirus 2 (SARS-CoV-2) or asymptomatic forms of coronavirus infection (COVID-19) in maintenance hemodialysis (HD) patients has not yet been clarified. The COVID-19 pandemic has been particularly severe in hemodialysis patients due to older age and comorbidities such as cardiovascular disease, diabetes, hypertension, obesity, and uremic syndrome, with a high estimated mortality of approximately 20% [1–4]. It has also been suggested that immune deregulation and immune senescence may contribute to the poor outcome in this population. Indeed, uremia has been found to induce impaired immunity [5]. Because of the high risk of mortality in this population and the threat of continued waves of the epidemic, we characterized the humoral response to SARS-CoV-2 infection in hemodialysis patients in two dialysis facilities and compared them to a population without kidney failure.

## 2. Materials and Methods

### 2.1. Populations and Design

This cross-sectional multicenter retrospective and observational study included patients on maintenance hemodialysis in the Clinique de l'Estrée and the Clinique Claude Bernard (two private health clinical centers).

Eligible patients were on maintenance hemodialysis during the first wave of the epidemic in France in March and April 2020 and were still alive 6 months later.

Patients in the two dialysis centers who were diagnosed as infected with SARS-CoV-2 after the first wave of the epidemic in March and April 2020, and those who arrived from another dialysis facility with an already positive diagnosis of SARS-CoV-2 infection before, during or after the first wave of the epidemic in March and April 2020, were excluded. The hemodialysis patients who came from another dialysis center or who began hemodialysis in the two dialysis centers after the wave of the epidemic in March and April 2020 were also excluded.

Two subpopulations were defined in the eligible population. The population of interest included patients with significant circulating levels of SARS-CoV-2 antibodies 6 months after first wave of the epidemic. The second group included patients from the population of interest who had been symptomatic and were considered to be infected with SARS-CoV-2 during the first wave of the epidemic. Diagnostic criteria were a positive nasopharyngeal swab RT-PCR assay or typical signs of the disease (fever, asthenia, cough, and dyspnea) with abnormal biological results (lymphopenia, elevated CRP, ferritin, troponin, or D-Dimers) or typical pulmonary features on imaging such as ground-glass opacity on a chest CT. Because asymptomatic infected hemodialysis patients were not screened during the epidemic wave and, therefore, did not undergo biological and radiological tests, only hemodialysis patients who were symptomatic and considered as infected during this period were investigated for clinical, biological, and radiological parameters. Additionally, a control population including healthcare workers without kidney failure and with positive serology 6 months after the first wave of the epidemic was established. These healthcare workers were nurses, care givers, housekeepers, and clinical physicians working during the same period of the study in one of the two clinics.

### 2.2. Ethics and Consent

All the data from the different populations were gathered from electronic records from the two dialysis units. Circulating levels of SARS-CoV-2 antibodies were obtained from blood samples with two dry tubes drawn before the beginning of the dialysis session in the hemodialysis patients and from venous blood samples for healthcare workers at the same time. All hemodialysis patients and healthcare workers enrolled in this study provided written informed consent to participate at this study and for the use of the data for research. Data collection was declared to the French Commission Nationale de l'informatique et des Libertés (CNIL) and registered as 2221881. This observational study was also approved by the Institutional Review Board (IRB).

### 2.3. Data Measurement and Collection

Circulating levels of SARS-CoV-2 antibodies were determined using a Roche Elecsys test. Elecsys® Anti-SARS-CoV-2 is an immunoassay for the qualitative in vitro detection of antibodies (including IgG and IgM) to SARS-CoV-2 in human and serum plasma. The assay uses a recombinant protein representing the nucleocapsid (N) antigen in a double-antigen sandwich format. Anti-SARS-CoV-2 detects antibody titers, which have been shown to correlate with neutralizing antibodies in neutralization assays [6]. Measurement of electrochemiluminescence intensity is directly correlated to the antibody titer and is expressed by a cutoff index (COI). COI < 1.0 is considered nonreactive, while COI ≥ 1.0 is reactive. Clinical, biological, and radiological data were only available in hemodialysis patients diagnosed as positive during the first wave of the epidemic and with a positive serology 6 months later. In hemodialysis patients with a positive serology but without a diagnosis of SARS-CoV-2 infection during the epidemic, only the age, gender, and circulating levels of SARS-CoV-2 antibodies were available. Diagnosis of SARS-CoV-2 infection during the epidemic was based on reverse transcription-polymerase chain reaction (RT-PCR) from nasopharyngeal swab samples, typical symptoms of the disease associated with biological results, or typical COVID-19 pulmonary lesions on the chest scan [7]. The nasopharyngeal swab for RT-PCR of SARS-CoV-2 was not performed in all 299 hemodialysis patients but only in patients with typical clinical symptoms of the disease. Clinical criterion was the need for oxygen therapy or not. The biological parameters evaluated were lymphocyte count, neutrophil-lymphocyte ratio, CRP, D-dimers, serum ferritin, troponin, and BNP plasma levels. The extent of pulmonary lesions was assessed by chest scan and classified in five degrees of severity according to the French Society of Thoracic Imaging recommendations with grading lung involvement as absent or minimal (<10%), moderate (<25%), extensive (25–50%) severe (50–75%), or critical (>75%) [8].

### 2.4. Objectives and Outcomes

The proportion of patients with a positive serology to SARS-CoV-2 antibodies (population of interest) was assessed in the eligible population, and the proportion of patients diagnosed six months earlier (symptomatic population) was determined in the population of interest. The humoral response to SARS-CoV-2 was evaluated by an analysis of the symptomatic population. In particular, the association between circulating levels of SARS-CoV-2 antibodies and biological parameters (BNP, D-dimers, lymphocyte count, ferritin, troponin, CRP, and neutrophil-lymphocyte ratio (NLR) and need for oxygen therapy and pulmonary extension were investigated. Clinical patient profiles were determined in the symptomatic population. Finally, circulating levels of SARS-CoV-2 antibodies were compared between the population of interest and the control population.

### 2.5. Statistical Analysis

Continuous variables were reported as medians and interquartile ranges (IQR). Categorical variables are reported as numbers, percentages, and 95% bilateral confidence interval (CI) calculated using the Wilson method. Correlations between circulating antibody levels and continuous variables were assessed using the Pearson correlation coefficient and 95% bilateral CI. Correlation coefficients were interpreted as weak (*r* < 0.3), moderate or mild (0.3 ≤ *r* < 0.7), or strong (*r* ≥ 0.7) [9]. Associations between circulating antibody levels and each categorical variable were assessed using the Wilcoxon–Mann–Whitney (2 levels variables) or Kruskal–Wallis (>2 levels variables) tests. Clinical patient profiles were determined using a clustering approach that handles missing data through multiple imputation and consensus clustering [10]. The variables used were circulating antibody levels, age, gender, need for oxygen therapy, pulmonary extension, BNP, D-dimers, lymphocyte count, ferritin, troponin, CRP, and NLR. Clustering was performed with 10 imputations and the k-mean and k-median algorithms. Results for continuous variables were reported as medians and IQR for the obtained clusters and compared using the Wilcoxon–Mann–Whitney test. Results for categorical variables were reported as numbers and percentages for the obtained clusters and compared using Fisher's exact test. Circulating antibody levels were compared between the population of interest and the control population using the Wilcoxon–Mann–Whitney test on data adjusted for age and sex (adjustment involves removing the effect of age and sex from the circulating antibody levels). *p* < 0.05 was considered to be statistically significant. All statistical analyses were performed using R Statistical Software (Version 4.0.3).

## 3. Results

**Figure 1:**
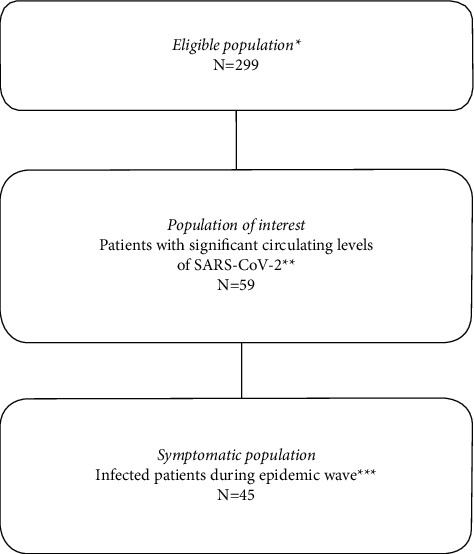
Flow chart of the different populations. ^*∗*^Patients in maintenance hemodialysis during the first wave of the epidemic, March–April 2020, and still living 6 months later. ^*∗∗*^6 months after the first wave of the epidemic. ^*∗∗∗*^RT-PCR positive (*n* = 25) or typical symptoms of the disease with biological abnormalities (*n* = 1) or typical pulmonary features on the chest scan (*n* = 19).

A total of 299 hemodialysis patients who were present in the two dialysis centers in March and April 2020 during the first wave of the epidemic and who were still alive 6 months later underwent a SARS-CoV-2 antibody assay (Figure 1). Fifty-nine (19.7% [15.6%; 24.7%]) of these hemodialysis patients were found to have a positive SARS-CoV-2 antibody serology 6 months later. This number was different from the 45 hemodialysis patients who were considered to be infected by SARS-CoV-2 during the first wave of the epidemic in March and April 2020. Thus, 14 patients (23.7% [14.7%; 36.2%]) were not detected during the first wave because of asymptomatic forms of SARS-CoV-2 infection. On the other hand, all of the hemodialysis patients who were diagnosed with SARS-CoV-2 infection from March to April 2020 developed a persistent humoral response with significant circulating levels of SARS-CoV-2 antibodies, 6 months later.

### 3.1. Characteristics of Symptomatic Hemodialysis Patients

The characteristics of the 45 hemodialysis patients who corresponded to the symptomatic population diagnosed as infected during the epidemic wave with a positive serology of SARS-CoV-2 antibodies 6 months later are illustrated in Table 1.

### 3.2. Association between Circulating Levels of SARS-CoV-2 Antibodies and Clinical and Biological Parameters

**Figure 2:**
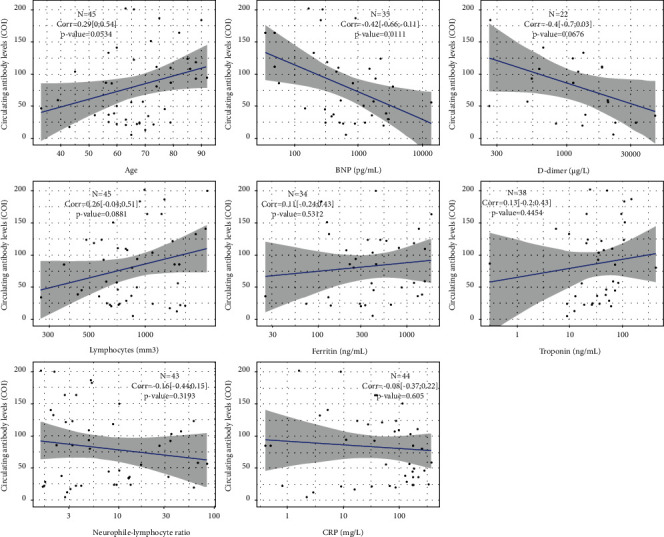
Values of Pearson correlation coefficient between the circulating levels of antibodies against SARS-CoV-2 and the different continuous variables (age and biological parameters) of the hemodialysis patients.

**Figure 3:**
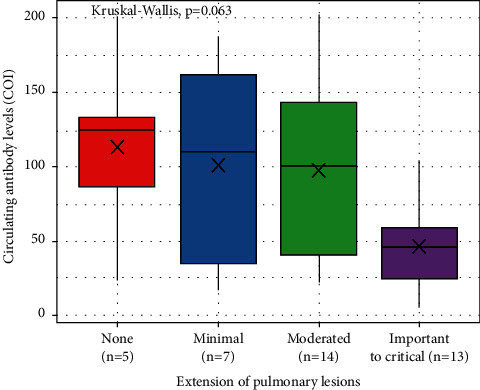
Comparison of the circulating levels of antibodies against SARS-CoV-2 in the different subgroups of hemodialysis patients according to the degree of pulmonary extension lesions by using the Kruskal–Wallis test.

Figure 2 shows the correlations between the circulating levels of SARS-CoV-2 antibodies, age, and biological parameters. Except for a moderate negative correlation with circulating levels of BNP (*r* = −0.42, 95% CI [−0.66; −0.11]), no significant correlations were observed between levels of circulating antibodies and these continuous variables. There was no significant difference in circulating levels of antibodies between men and women or between the group with and without oxygen therapy. However, as shown in Figure 3, there was a trend towards a decrease in circulating levels of antibodies in the subgroup of hemodialysis patients with the more extensive pulmonary lesions than in those without or with slight pulmonary lesions.

### 3.3. Clinical Patient Profiles

After imputation of missing data, the 45 hemodialysis patients diagnosed as infected during the first wave of the epidemic were grouped into 2 clusters according to their characteristics (Table 2).

Cluster 1 (*N* = 21) had significantly higher median circulating levels of SARS-CoV-2 antibodies than cluster 2 (*N* = 24) with median values of 122 [23.1; 164] and 48.5 [33; 87.6], respectively (*p*=0.029). Comparison of the two clusters showed significantly higher circulating levels of BNP in cluster 2 (with lower circulating levels of SARS-CoV-2 antibodies) than in cluster 1 (with higher circulating levels of SARS-CoV-2 antibodies) with values of 1013 pg/mL [406.5; 1944.8] and 259 pg/mL [111.5; 527], respectively (*p*=0.004). There was a statistically significant increase in circulating levels of CRP in cluster 2 (with lower circulating levels of SARS-CoV-2 antibodies) than in cluster 1 (with higher circulating levels of SARS-CoV-2 antibodies) with 174 mg/L [87.4; 198.5] and 15.9 mg/L [3.8; 49.8], respectively (*p* < 0.0001). Conversely, the lymphocyte count was significantly lower in cluster 2 than in cluster 1, with 706/mm^3^ [574.5; 842.8] and 1274/mm^3^ [902; 1531], respectively (*p*=0.0001). PNLR was also significantly different with 3.2 [2.1; 4] and 13.2 [8.8; 35.5], in clusters 1 and 2, respectively (*p* < 0.0001). There were no statistical differences between the two clusters in other continuous variables such as age or biological parameters such as ferritin, D-dimers, and troponin. Differences in categorical variables such as oxygen therapy and the extent of pulmonary lesions were also statistically significant between the two clusters. The proportion of hemodialysis patients requiring oxygen was significantly higher in cluster 2 (with lower circulating levels of SARS-CoV-2 antibodies) than in cluster 1 (with higher circulating levels of SARS-CoV-2 antibodies) with 78.3% (18) and 19% (4), respectively (*p*=0.0002). The percentage of hemodialysis patients with severe pulmonary lesions was also significantly higher in cluster 2 than in cluster 1 (*p*=0.0002). No statistical difference was observed for gender.

### 3.4. Comparison of Circulating Levels of SARS-CoV-2 Antibodies in Hemodialysis and Control Groups

**Figure 4:**
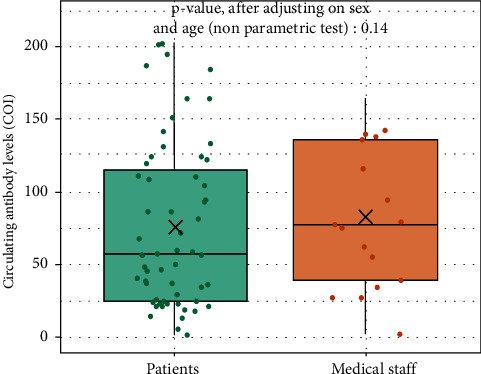
Comparison of the circulating levels of SARS-CoV-2 antibodies between the hemodialysis patients (*N* = 57) and the control group of healthcare workers without kidney disease (*N* = 17).

The characteristics of hemodialysis patients and controls are summarized in Table 3.  Figure 4 shows no statistical difference between the group of hemodialysis patients and the group of healthcare workers with a median (IQR) of 57.1 [24.7.4; 115.0] in the hemodialysis group and 77.1 (39.0; 136) in the control group (*p*=0.014).

### 3.5. Discussion/Conclusions

The main finding of this study is the presence of a persistent humoral response 6 months later in all surviving hemodialysis patients who were diagnosed with SARS-CoV-2 infection in March and April 2020. The other finding is that clinical, radiological, and several biological parameters reflecting disease severity were significantly higher in the hemodialysis patients with lower circulating levels of SARS-CoV-2 antibodies than in the group with higher circulating levels of SARS-CoV-2 antibodies. This study also shows that circulating levels of SARS-CoV-2 antibodies observed 6 months after infection in hemodialysis patients were not statistically different from those in the control group of healthcare workers.

This study shows that the serological prevalence of SARS-CoV-2 antibodies in hemodialysis patients is 19.73% [15.62%; 24.65%]. This proportion is much higher than that in the French national cohort of dialysis patients, with a general prevalence of 3.3% with maximum values of 10% and 9% in Alsace and Ile-de-France regions, respectively [1]. The difference in prevalence between our results and the French cohort study may have several explanations. First, the diagnostic criteria in the French cohort study did not include an antibody assay and, thus, may have not identified and may have underestimated subclinical, asymptomatic cases. If hemodialysis patients identified by antibody assays had not been included in our study, the proportion of hemodialysis patients infected by SARS-CoV-2 decreased from 19.7% (59 patients out of 299) to 15.0% (45 patients out of 299). The French cohort study may also have underestimated the number of cases due to less-accurate reporting results in certain areas of France infected by SARS-CoV-2 in the study from the French REIN registry [1]. Indeed, the Seine Saint Denis department in the suburb of Paris was much more severely affected by the epidemic than the rest of the Ile-de-France region around Paris. The percentage of undetected hemodialysis patients infected by SARS-CoV-2 in our study was lower than that in previous studies. Indeed, the percentage of undetected hemodialysis patients was 23.73% in our study compared to 40.3% and 47.5% in an English and Chinese study, respectively [11, 12]. Nonetheless, similarly to our study where all hemodialysis patients diagnosed as infected developed secondarily SARS-CoV-2 antibodies, the proportion of the hemodialysis patients diagnosed as infected in the English study who secondarily develop SARS-CoV-2 antibodies was 97% [11]. This antibody response is similar to that found in patients without kidney disease and in the general population [13, 14].

The second result of this study is that unlike what might be expected, there was no positive correlation between the biological, clinical, and radiological surrogate markers of the severity of COVID-19 and circulating levels of antibodies in hemodialysis patients infected during the first wave of the epidemic. Indeed, a previous study that compared the kinetics of SARS-CoV-2-specific IgM and IgG responses in COVID-19 patients suggests that the antibody response was more significant in intensive unit care (ICU) patients than in non-ICU patients during the acute phase of the SARS-CoV-2 infection [15]. Another study performed to obtain potential plasma donors showed that factors such as sex, age, and hospitalization can be used to identify individuals with a high probability of a strong antibody response [16]. This cross-sectional study was also limited because it was performed during the acute period of the infection and only represents the antibody response at one time point [16]. However, symptomatic COVID-19 in hemodialysis patients was also found to confer a stronger SARS-CoV-2 antibody response than asymptomatic disease [17]. In contrast, in our study, there was a trend towards a negative correlation between the concentration of circulating antibodies and the surrogate markers related to disease severity, which was confirmed when the clustering procedure was performed in the hemodialysis population.

Finally, the similar concentrations of circulating SARS-CoV-2 antibodies found 6 months after SARS-CoV-2 infection between the hemodialysis patient group and the control group of healthcare workers without kidney disease, even after adjustment for age and gender, may seem surprising, since hemodialysis patients are known to have an impaired humoral response to vaccination and infection [13]. However, the antibody response to SARS-CoV-2 infection in hemodialysis patients compared to the nondialysis population remains a subject of debate [13, 18]. Although the results of our study provide some insight on this subject, they should be extrapolated with caution due to the small number of patients in the two groups and the possibility of cofounding factors. Nevertheless, they are consistent with previous, smaller studies that identified a similar humoral response after SARS-CoV-2 infection in hemodialysis and nonhemodialysis patients [13]. Recently, Bruno et al. reported that hemodialysis patients, patients with kidney failure, and kidney transplant recipients could produce significant and satisfying circulating levels of SARS-CoV-2 antibodies even if the viral clearance appeared to be longer than in the general population [19]. The humoral immune response to SARS-CoV-2 6 months after the infection was also found to be durable in symptomatic hemodialysis patients and not lower than in symptomatic healthcare staff [17].

Our study has certain limitations including the lack of kinetic of antibody response over time between the beginning of the infection and the measurement of circulating SARS-CoV-2 antibodies 6 months later. Thus, unlike in other longitudinal studies, there is no information about the rate of decrease in the circulating antibodies over time [20]. However, the strength of this study is to show that all the hemodialysis patients who were diagnosed with SARS-CoV-2 infection during the epidemic in March and April 20020 developed sustained and persistent significant circulating levels of SARS-CoV-2 antibodies for at least 6 months.

In conclusion, this study shows that circulating levels of antibodies against SARS-CoV-2 are still present 6 months later in all hemodialysis patients infected six months before [11]. We also show that, unlike what might be expected, there was no positive correlation or association between the severity of disease, assessed by biological parameters, oxygen requirements, and pulmonary extension and elevated circulating levels of antibodies [13, 14]. On the contrary, a significant association was observed between several clinical, biological, and radiological criteria of disease severity in the cluster with lower circulating levels of antibodies using an unsupervised classification procedure. Furthermore, there was no significant difference between circulating antibody levels in a control group of healthcare workers who were infected at the same period and patients on hemodialysis.

## Figures and Tables

**Table 1:** Characteristics of symptomatic patients on maintenance hemodialysis (*N* = 45) diagnosed as infected with SARS-CoV-2 from March to April of the epidemic and who were still alive 6 months later.

Variables	Total patients, *N* = 45 (%)	Values median [IQR^1^]
Age (years), median [IQR]	45 (100)	67.0 [59.0; 77.0]

*Sex*		
Women	18 (40)	
Men	27 (60)	

*Oxygen supply*		
Yes	22 (50)	
No	22 (50)	

*Chest scan* ^*∗*^	39 (87)	
No pulmonary injury	5 (11)	
Minimal pulmonary injury (<10%)	7 (16)	
Moderate pulmonary injury (10–25%)	14 (31)	
Extensive to critical pulmonary injury (>25%)	13 (29)	

*Biological parameters*		
Lymphocyte nadir (1000–4800/mm^3^^*∗∗*^), median [IQR]	45 (100)	840.0 [642.0; 1274.0]
Ferritin peak (30–400 ng/mL^*∗∗*^), median [IQR]	34 (76)	409.0 [233.2; 860.2]
D-dimer peak (<500 ng/mL^*∗∗*^), median [IQR]	22 (49)	1276.0 [708.0; 1964.2]
CRP peak (<5 mg/L^*∗∗*^), median [IQR]	44 (98)	85.0 [14.8; 178.5]
BNP (100–400 pg/mL^*∗∗*^), median [IQR]	35 (78)	534.0 [286.5; 1570.5]
Troponin (<34.2 ng/mL^*∗∗*^), median [IQR]	38 (84)	38.8 [20.8; 72.0]
Neutrophil/lymphocyte ratio (<5^*∗∗*^), median [IQR]	43 (96)	5.0 [2.8; 13.2]

^1^IQR: interquartile range. ^*∗*^Pulmonary extension was usually classified by five degrees of severity according to the French Society of Thoracic Imaging recommendations by grading lung involvement as absent or minimal (<10%), moderate (<25%), extensive (25–50%), severe (50–75%), and critical (>75%) (8). Because of the low number of hemodialysis patients with severe pulmonary injuries, extensive, severe, and critical involvement were gathered into one (>25%). ^*∗∗*^Normal values.

**Table 2:** Comparison of categorial and continuous variables between cluster 1 and cluster 2 characterized by statistical differences in circulating levels of SARS-CoV-2 antibodies.

	Cluster 1 (*N* = 21)	Cluster 2 (*N* = 24)	*p* value
Circulating levels of antibodies	122 [23.1/164]	48.5 [33/87.6]	0.029^*∗*^
Age	68 [60/79]	65 [57.8/74.8]	0.45^*∗*^
BNP (pg/mL)	259 [111.5/527]	1013 [406.5/1944.8]	0.004^*∗*^
D-dimers (pg/mL)	1115 [750/1288.5]	1360 [865/2130.5]	0.24^*∗*^
Lymphocytes (/mm^3^)	1274 [902/1531]	706 [574.5/842.8]	0.0001^*∗*^
Ferritin (ng/mL)	362 [182/624]	409[274.5/1039.5]	0.75^*∗*^
Troponin (ng/mL)	33,5 [17.3/100.2]	41.2 [23.4/66.5]	0.77^*∗*^
Neutrophil/lymphocyte ratio	3.2 [2.1/4]	13.2 [8.8/35.5]	<0.0001^*∗*^
CRP (mg/L)	15.9 [3.8/49.8]	174 [87.4/198.5]	<0.0001^*∗*^
*Gender*			
Women	8 (38.1%)	10 (41.7%)	1.00^*∗∗*^
Men	13 (61.9%)	14 (58.3%)	
*Need oxygen*			
Yes	4 (19%)	18 (78.3%)	0.0002^*∗∗*^
No	17 (81%)	5 (21.7%)	
*Pulmonary lesion extension*			
Nothing	5 (27.8%)	0 (0%)	0.0002^*∗∗*^
Mild	6 (33.3%)	1 (4.8%)	
Moderate	6 (33.3%)	8 (38.1%)	
Serious to critical	1 (5.6%)	12 (57.1%)	

^*∗*^Wilcoxon–Mann–Whitney test. ^*∗∗*^Fisher's exact test.

**Table 3:** Characteristics of the hemodialysis patients (*N* = 59) and the control group without kidney disease (*N* = 17).

	Hemodialysis patients, *N* = 59	Controls, *N* = 17
Age (years), median [IQR]	65.0 [57.0–73.5]	39.0 [31.0–48.0]
Sex, *n* (%)		
Women	21 (36)	12 (71)
Men	38 (64)	5 (29)

## Data Availability

All the data can be made available on request at the ECTEN-EUROPEAN CLINICAL TRIAL EXPERTS NETWORK.
